# Supplementary material for: Evaluation of rice wild relatives as a source of traits for adaptation to iron toxicity and enhanced grain quality
Source: PLoS One. 2020 Jan 3;15(1):e0223086. doi: 10.1371/journal.pone.0223086 (PMC6941827; doi:10.1371/journal.pone.0223086)
Supplement: S1 Table — (DOCX) [file pone.0223086.s001.docx]

Supplementary Table S1: List of germplasm used in the screening experiment

| **IRGC Acc. No.** | **Species** | **Origin** | **Genome type** | **Tolerance ranking** |
| --- | --- | --- | --- | --- |
| [NI] | *O. sativa* (IR 72) | Indica x japonica cross (IRRI) | AA | Sensitive |
| [NI] | *O. sativa* (IR 29) | Indica x japonica cross (IRRI) | AA | Sensitive |
| [NI] | *O. sativa* (DOM SOFID) | Iran (GWAS-Panel) | AA | Tolerant |
| [NI] | *O. sativa* (Vary Vato 462) | Madagascar (GWAS-Panel) | AA | Tolerant |
| [NI] | *O. sativa* (Tchibanga) | Gabon (GWAS-Panel) | AA | Tolerant |
| [NI] | *O. sativa* (Taichung native 1) | China (GWAS-Panel) | AA | Tolerant |
| [NI] | *O. sativa* (Suakoko 8) | Liberia (AfricaRice) | AA | Tolerant |
| [NI] | *O. sativa* (Kitrana 508) | Madagascar (GWAS-Panel) | AA | Tolerant |
| [NI] | *O. sativa* (Kiang-Chan-Chin) | China (GWAS-Panel) | AA | Sensitive |
| 122403 | *O. sativa* (IRRI 154) | Bangladesh (IRRI) | AA | Unknown |
| [NI] | *O. sativa* (Guan Yin Tsan) | China (GWAS-Panel) | AA | Sensitive |
| [NI] | *O. sativa* (FL 483) | India (Mapping population) | AA | Tolerant |
| [NI] | *O. sativa* (Yodanya) | Myanmar (GWAS-Panel) | AA | Intermediate |
| 126953 | *O. sativa* (Curinga) | [NI] (IRRI) | AA | Unknown |
| [NI] | *O. sativa* (BW 348) | Togo (AfricaRice) | AA | Tolerant |
| [NI] | *O. sativa* (Arabi) | Sri Lanka (GWAS-Panel) | AA | Tolerant |
| [NI] | *O. glaberrima*(CG 14) | Guinea (AfricaRice) | AA | Tolerant |
| 88428 | *O. spontanea* | Bangladesh | AA | Unknown |
| 81805 | *O. spontanea* | Guyana | AA | Unknown |
| 80612 | *O. spontanea* | India | AA | Unknown |
| 104620 | *O. spontanea* | China | AA | Unknown |
| 88785 | *O. rufipogon* | Bangladesh | AA | Unknown |
| 81801 | *O. rufipogon* | Indonesia | AA | Unknown |
| 126954 | *O. rufipogon* | Malaysia | AA | Unknown |
| 106276 | *O. rufipogon* | Papua New Guinea | AA | Unknown |
| 106123 | *O. rufipogon* | India | AA | Unknown |
| 105491 | *O. rufipogon* | Malaysia | AA | Unknown |
| 105293 | *O. rufipogon* | Australia | AA | Unknown |
| 103823 | *O. rufipogon* | China | AA | Unknown |
| 88868 | *O. nivara* | Cambodia | AA | Unknown |
| 103837 | *O. nivara* | Bangladesh | AA | Unknown |
| 102463 | *O. nivara* | Bangladesh | AA | Unknown |
| 101510 | *O. nivara* | India | AA | Unknown |
| 100916 | *O. nivara* | China | AA | Unknown |
| 100593 | *O. nivara* | Chinese Taipei (Taiwan) | AA | Unknown |
| 104137 | *O. barthii x glaberrima* | Cameroon | AA | Unknown |
| 89146 | *O. barthii* | Zambia | AA | Unknown |
| 106238 | *O. barthii* | Mali | AA | Unknown |
| 105613 | *O. barthii* | Botswana | AA | Unknown |
| 103895 | *O. barthii* | Senegal | AA | Unknown |

| **IRGC Acc. No.** | **Species** | **Origin** | **Genome type** | **Tolerance ranking** |
| --- | --- | --- | --- | --- |
| 103581 | *O. barthii* | Chad | AA | Unknown |
| 89153 | *O. longistaminata* | Zambia | AA | Unknown |
| 101431 | *O. longistaminata* | Tanzania | AA | Unknown |
| 101206 | *O. longistaminata* | Benin | AA | Unknown |
| 113666 | *O. longistaminata* | Namibia | AA | Unknown |
| 82041 | *O. meridionalis* | Australia | AA | Unknown |
| 105564 | *O. meridionalis* | Indonesia | AA | Unknown |
| 105290 | *O. meridionalis* | Australia | AA | Unknown |
| 82031 | *O. glumaepatula* | Brazil | AA | Unknown |
| 105692 | *O. glumaepatula* | Brazil | AA | Unknown |
| 100184 | *O. glumaepatula* | Cuba | AA | Unknown |
| 81794 | *O. officinalis* | Indonesia | CC | Unknown |
| 102460 | *O. officinalis* | Bangladesh | CC | Unknown |
| 86466 | *O. rhizomatis* | Sri Lanka | CC | Unknown |
| 89245 | *O. eichingeri* | Uganda | CC | Unknown |
| 81803 | *O. eichingeri* | Sri Lanka | CC | Unknown |
| 105322 | *O. malampuzhaensis* | India | BBCC | Unknown |
| 83825 | *O. minuta* | Papua New Guinea | BBCC | Unknown |
| 105082 | *O. punctata* | Philippines | BB, BBCC | Unknown |
| 101429 | *O. punctata* | Uganda | BB, BBCC | Unknown |
| 101409 | *O. punctata* | Ghana | BB, BBCC | Unknown |
| 100886 | *O. punctata* | India | BB, BBCC | Unknown |
| 105176 | *O. latifolia* | Malaysia | CCDD | Unknown |
| 105145 | *O. latifolia* | Colombia | CCDD | Unknown |
| 104965 | *O. alta* | China | CCDD | Unknown |
| 100161 | *O. alta* | Brazil | CCDD | Unknown |
| 101405 | *O. grandiglumis* | Brazil | CCDD | Unknown |
| 86527 | *O. australiensis* | Australia | EE | Unknown |
| 105147 | *O. longiglumis* | Indonesia | HHJJ | Unknown |
| 106469 | *O. granulata* | Viet Nam | GG | Unknown |
| 106445 | *O. granulata* | India | GG | Unknown |
| 81950 | *O. brachyantha* | Zambia | FF | Unknown |
| 104155 | *O. brachyantha* | Cameroon | FF | Unknown |
| 101236 | *O. brachyantha* | Mali | FF | Unknown |
| 101233 | *O. brachyantha* | Sierra Leone | FF | Unknown |

Tolerance ranking was based on Wu et al. (2014), Matthus et al (2015), or Sikirou et al. (2016).
