# Supplementary material for: Evaluation of rice wild relatives as a source of traits for adaptation to iron toxicity and enhanced grain quality
Source: PLoS One. 2020 Jan 3;15(1):e0223086. doi: 10.1371/journal.pone.0223086 (PMC6941827; doi:10.1371/journal.pone.0223086)
Supplement: S2 Table — (DOCX) [file pone.0223086.s002.docx]

Supplementary Table S2: Comparison of mean values and variability of selected traits of domesticated and wild rice species grown in control conditions, acute Fe stress, or chronic Fe stress

| Trait | Domesticated rice | | | | |  | Rice wild relatives | | | | Difference |
| --- | --- | --- | --- | --- | --- | --- | --- | --- | --- | --- | --- |
|  | Mean value | Count | | Range | CV |  | Mean value | Count | Range | CV |  |
| LBS acute stress | 0.30 | | 17 | 0.13-0.58 | 0.41 |  | 0.44 | 48 | 0.10-1.41 | 0.69 | ns |
| LBS chronic stress | 0.84 | | 17 | 0.28-2.32 | 0.53 |  | 1.24 | 49 | 0.50-4.33 | 0.61 | * |
| Leaf Fe concentration control (mg kg^-1^) | 339 | | 8 | 151-724 | 0.62 |  | 214 | 9 | 71-460 | 0.57 | ns |
| Leaf Fe concentration acute stress (mg kg^-1^) | 807 | | 8 | 372-2868 | 1.04 |  | 774 | 9 | 169-2683 | 0.98 | ns |
| Leaf Fe concentration chronic stress (mg kg^-1^) | 975 | | 8 | 651-1354 | 0.27 |  | 1356 | 9 | 494-2320 | 0.52 | ns |
| Grain yield control (g plant^-1^) | 22.2 | | 16 | 3.7-49.3 | 0.62 |  | 21.4 | 7 | 0.1-58.6 | 1.03 | ns |
| Grain yield acute stress (g plant^-1^) | 26.3 | | 16 | 0.8-58.0 | 0.62 |  | 21.1 | 10 | 0.04-68.4 | 1.04 | ns |
| Grain yield chronic stress (g plant^-1^) | 19.9 | | 15 | 2.3-41.6 | 0.69 |  | 18.5 | 7 | 0.3-61.6 | 1.18 | ns |
| Straw yield control (g plant^-1^) | 37.8 | | 17 | 17.0-71.1 | 0.45 |  | 60.8 | 55 | 1.1-243.8 | 0.87 | ns |
| Straw yield acute stress (g plant^-1^) | 53.6 | | 17 | 22.3-102.8 | 0.48 |  | 80.3 | 46 | 2.5-444.9 | 0.91 | ns |
| Straw yield chronic stress (g plant^-1^) | 45.8 | | 17 | 18.3-84.3 | 0.50 |  | 69.4 | 48 | 2.1-368.5 | 0.91 | ns |
| Spikelet sterility control (%) | 42.0 | | 16 | 2.3-97.4 | 0.50 |  | 47.4 | 7 | 3.4-96.0 | 0.54 | ns |
| Spikelet sterility acute stress (%) | 47.7 | | 16 | 4.1-99.5 | 0.49 |  | 44.9 | 10 | 5.6-96.3 | 0.54 | ns |
| Spikelet sterility chronic stress (%) | 53.8 | | 15 | 13.8-92.5 | 4.42 |  | 47.6 | 7 | 7.6-97.0 | 0.49 | ns |

LBS leaf bronzing score; CV coefficient of variation (standard deviation / mean value). Count indicates the number of genotypes included in each of the analyses; it varies between traits because not all traits were measured in all genotypes. The ‘Difference’ column indicates whether the mean values between domesticated and wild rice genotypes differed significantly by t-Test; ns not significant; *P<0.05.
